# Fecal Short-Chain Fatty Acid Concentrations Increase in Newly Paired Male Marmosets (Callithrix jacchus)

**DOI:** 10.1128/mSphere.00794-20

**Published:** 2020-09-16

**Authors:** Lifeng Zhu, Mallory J. Suhr Van Haute, Haley R. Hassenstab, Caroline Smith, Devin J. Rose, Aaryn C. Mustoe, Andrew K. Benson, Jeffrey A. French

**Affiliations:** a Department of Biology, University of Nebraska at Omaha, Omaha, Nebraska, USA; b Nebraska Food for Health Center, University of Nebraska-Lincoln, Lincoln, Nebraska, USA; c Department of Psychology, University of Nebraska at Omaha, Omaha, Nebraska, USA; d Department of Food Science and Technology, University of Nebraska-Lincoln, Lincoln, Nebraska, USA; University of Michigan-Ann Arbor

**Keywords:** common marmosets, cohabitation and social contact, short-chain fatty acids, sex difference

## Abstract

The role by which the gut microbiome influences host health (e.g., energy equilibrium and immune system) may be partly mediated by short-chain fatty acids, which are bacterial fermentation products from the dietary fibers. However, little is known about longitudinal changes in gut microbiome metabolites during cohabitation alongside social contact. In common marmosets (Callithrix jacchus), the gut microbiome community is influenced by social contact, as newly paired males and females develop convergent microbial profiles. Here, we monitored the dynamics of short-chain fatty acid concentrations in common marmoset feces from the prepairing (PRE) to postpairing (POST) stages. In males, we observed that the concentrations of acetate, propionate, isobutyrate, and isovalerate significantly increased in the POST stage compared to the PRE stage. However, no significant changes were found in females. We further found that the propionate concentration was significantly positively correlated with the abundance of *Phascolarctobacterium* in the male feces. Thus, the sex difference in the changes in the concentrations of short-chain fatty acids might be related to sex-biased gut microbiome transmission after pairing. We suggest that the significant changes in the gut microbiomes and some short-chain fatty acids of the common marmoset during cohabitation may contribute to physiological homeostasis during pairing.

**IMPORTANCE** This study addressed a knowledge gap about longitudinal changes in the gut microbiome metabolites during animal pairing. This research in the laboratory common marmoset can control for the confounding factors such as diet and other environmental conditions. *Phascolarctobacterium* showed the highest contribution to the sex-biased transmission of the female to the male after pairing. Here, we observed the sex difference in the increase in short-chain fatty acid concentration in the feces of newly paired marmosets, which may be caused by the sex-biased gut microbiome transmission after pairing.

## Abstract (precis)

This study addressed a knowledge gap about longitudinal changes in the gut microbiome metabolites during animal pairing. This research in the laboratory common marmoset can control for the confounding factors such as diet and other environmental conditions. *Phascolarctobacterium* showed the highest contribution to the sex-biased transmission of the female to the male after pairing. Here, we observed the sex difference in the increase in short-chain fatty acid concentration in the feces of newly paired marmosets, which may be caused by the sex-biased gut microbiome transmission after pairing.

## OBSERVATION

Marmosets (Callithrix jacchus) are family-living primates, with groups consisting of adult males and females, who form long-term socially monogamous and cooperative breeding relationships, and their offspring. The principal bacteria in the marmoset gut microbiome include *Firmicutes* (∼39%), *Bacteroidetes* (∼29%), and *Actinobacteria* (∼27%) (1). We have previously reported that newly paired marmosets displayed a significant convergence in their gut microbiomes during the first 8 weeks of cohabitation: significantly increase in the abundance of five genera (*Phascolarctobacterium*, *Alloprevotella*, *Anaerobiospirillum*, *Sutterella*, and *Coprobacter*) in both male and female feces after pairing (1). This finding may be associated with their social contact and affiliative behavior (e.g., social grooming, genital investigation, social approach, and mounts and copulations) (2–4). In addition, considering the pairing experiment performed at the same time, the unknown factors other than pairing could also have affected gut microbiome changes.

*Phascolarctobacterium* showed the highest contribution in the gut microbiome transmission in the newly paired marmosets, and this microbiome group exhibited the highest contribution to the sex-biased transmission pattern of the female to the male (1). Some studies have suggested that the transmitted gut microbiome within primate social groups may be beneficial to the host health, but with no direct evidence (5, 6).

The gut microbiome plays an important role in host immune development and health (7, 8). Short-chain fatty acids (SCFAs) are the end products of fermentation of dietary fibers and protein by the gut microbiome (9–12). SCFAs (including acetate [ACE], propionate [PA], butyrate [BUTY], isobutyrate [ISOB], and isovalerate [ISOV]) have a key role in the maintenance of intestinal homeostasis and help maintain energy equilibrium, immune system function, and health (9–12). For example, PA can increase host serotonin (5-hydroxytryptamine) biosynthesis in the intestine and affect gut motility and hemostasis (10). PA also reduces host inflammation, enhances tissue insulin sensitivity, maintains homeostasis of glucose, and induces hormone release (e.g., of leptin) (11, 12).

*Phascolarctobacterium* can produce short-chain fatty acids, such as propionate (PA) (13). Based on the significant elevation in *Phascolarctobacterium* following social pairing in marmosets, we hypothesized that these changes in gut microbiome community following pairing would result in significant increases in production of SCFAs, especially PA.

**FIG 1:**
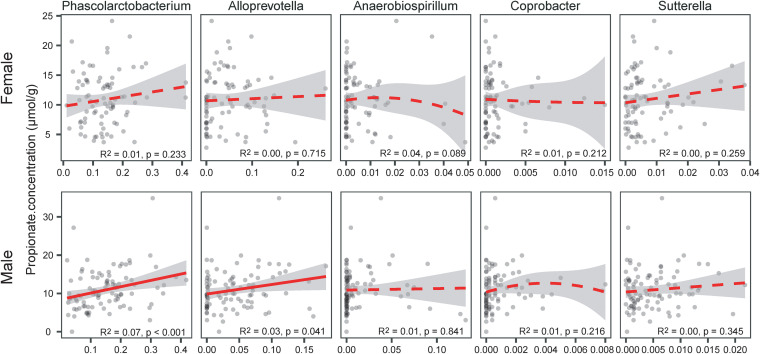
Concentrations of propionate (μmol/g, *y* axis) and the relative abundance of five genera *(Phascolarctobacterium*, *Alloprevotella*, *Anaerobiospirillum*, *Sutterella*, and *Coprobacter*). Dashed line, significant correlation based on the uncorrected *P* value. Solid line, nonsignificant correlation based on the uncorrected *P* value. The propionate concentration was significantly associated only with the abundance of *Phascolarctobacterium* in fecal samples from males after Benjamini-Hochberg correction (α = 0.05).

We measured the concentration of five SCFAs (acetate [ACE], propionate [PA], butyrate [BUTY], isobutyrate [ISOB], and isovalerate [ISOV]) from 228 fecal samples. The main SCFAs in these samples were ACE (mean: 44.91 ± 1.369 μmol/g), PA (mean: 10.86 ± 0.331 μmol/g), and BUTY (mean: 2.75 ± 0.151 μmol/g). The concentrations of the other two SCFAs were very low (ISOB: 0.35 ± 0.298 μmol/g; ISOC: 0.41 ± 0.341 μmol/g). We compared the SCFA concentration from prepairing (PRE) to postpairing (POST) for each sex. In males, we observed that the concentrations of ACE, PA, ISOB, and ISOV significantly increased in the POST stage compared to the PRE stage. No significant changes were found in female fecal samples after pairing, but the changes in PA after pairing were at the marginally significant level (Table 1, Benjamini-Hochberg [B-H] correction). Although there were significant increases in the abundance of five genera (*Phascolarctobacterium*, *Alloprevotella*, *Anaerobiospirillum*, *Sutterella*, and *Coprobacter*) in fecal samples from both males and females, the PA concentration was significantly associated only with the abundance of *Phascolarctobacterium* in fecal samples from male marmosets (Fig. 1, B-H correction). Therefore, we speculated that the highest contribution of *Phascolarctobacterium* to the sex-biased transmission pattern of the female to the male after pairing might result in the sex differences in the abundance changes of some SCFA concentrations.

**TABLE 1:** Average concentrations of the main short-chain fatty acids between PRE and POST stages^*a*^

Stage	Female						Male					
	Pair	Avg concn (μmol/g) of SCFA:					Pair	Avg concn (μmol/g) of SCFA:				
		ACE	PA	BUTY	ISOB	ISOV		ACE	PA	BUTY	ISOB	ISOV
PRE	P1	32.276	6.589	2.521	0.372	0.511	P1	43.541	8.295	2.583	0.405	0.574
	P2	33.776	11.238	1.110	0.229	0.301	P2	38.163	10.448	4.359	0.044	0.106
	P3	30.114	9.722	1.059	0.673	0.730	P3	41.156	14.046	3.994	0.547	0.596
	P4	32.658	8.962	0.618	0.062	0.019	P4	41.897	11.548	1.786	0.283	0.283
	P6	47.386	10.619	4.511	0.256	0.383	P6	30.623	6.957	0.849	0.099	0.123
	P7	39.489	9.689	2.639	0.273	0.323	P7	39.194	8.065	1.515	0.376	0.211
	P8	44.570	6.651	3.818	0.153	0.361	P8	35.155	8.191	0.810	0.126	0.098
	P9	40.672	7.107	2.040	0.000	0.043	P9	38.607	2.172	2.871	0.000	0.027

POST	P1	35.469	12.332	1.768	0.607	0.644	P1	50.912	10.543	2.908	0.520	0.662
	P2	43.088	13.829	2.903	0.248	0.288	P2	55.097	10.527	3.417	0.345	0.532
	P3	20.327	8.337	0.928	0.158	0.195	P3	54.263	18.076	3.102	0.479	0.510
	P4	41.029	11.197	2.116	0.605	0.657	P4	48.224	13.614	3.638	0.384	0.476
	P6	73.356	15.450	7.412	0.400	0.459	P6	49.268	11.374	2.276	0.495	0.224
	P7	50.789	12.073	3.576	0.546	0.660	P7	38.472	11.733	1.148	0.501	0.538
	P8	59.063	12.432	5.889	0.142	0.370	P8	33.851	11.686	2.135	0.469	0.513
	P9	47.334	12.822	2.552	0.517	0.616	P9	54.085	7.554	3.210	0.313	0.489

Wilcoxon paired test		0.078^NS^	0.016^NS^	0.055^NS^	0.148^NS^	0.195^NS^		0.039*	0.008*	0.461^NS^	0.016*	0.016*

a The main short-chain fatty acids in this study include acetate (ACE), propionate (PA), butyrate (BUTY), isobutyrate (ISOB), and isovalerate (ISOV). Wilcoxon paired test, uncorrected *P* value based on Wilcoxon paired test. *, significance after Benjamini-Hochberg correction (α = 0.05) within each sex. NS, nonsignificance after Benjamini-Hochberg correction (α > 0.05) within each sex.

*Phascolarctobacterium* bacteria are Gram-negative, obligately anaerobic, and non-spore-forming environmental microorganisms and have been found in soil, water, and some mammal feces (e.g., koala, human, and nonhuman primate) (1, 13–15). One of the main end products of the fermentation in *Phascolarctobacterium* is PA (13), which may have beneficial effects on the host (10–12, 16–18). For example, *Phascolarctobacterium* is positively correlated with the human positive mood (17), and *Phascolarctobacterium* has beneficial effects on host health and putatively decreases susceptibility to hepatic steatosis based on a rat model of nonalcoholic fatty liver (18). Moreover, PA is the essential mediator in the link between host nutrition, symbiotic gut microbiomes, and host physiology (19, 20). The purpose of physiological homeostasis is to neutralize or repair disturbance and to maintain stability (e.g., blood glucose level and blood pressure) (21). In addition, several diseases in humans (e.g., diabetes, obesity, and autoimmune disorders) are associated with reduced SCFA production in the gut (22–25).

Interestingly, the establishment of social bonds within pairmates (e.g., pair bonding) is important for maintaining physiological homeostasis (26). The data presented here extend these findings to the sex difference in changes in the fermentation metabolites of the gut microbiome during pair formation. Although the fecal SCFAs do not entirely reflect the total intestinal production (19, 20), examining SCFAs in common marmoset feces is a possible way (noninvasive method) to show SCFA metabolism in the intestine indirectly. We suggest that the gut microbiome metabolites in marmosets may have effects on the host physiology after pairing. Therefore, this study provides the new insight that one of the putative mechanisms between social bonding and physiological homeostasis in humans and nonhuman primates might involve microbial transmission in the gut and the associated metabolites under the condition of sex difference.

### Newly paired marmoset experiment.

We examined fecal short-chain fatty acid concentrations in eight adult common marmoset pairs (eight females and eight males, aged 1.5 to 7.5 years). Baseline fecal samples (PRE; 59 fecal samples) were collected during a 2-week period prior to pairing (PRE), during which marmosets resided with an opposite-sex partner or in a family group (1). Marmosets were then rehoused in a new enclosure with a new opposite-sex partner who was a previously unfamiliar and unrelated marmoset. Fecal samples were collected in the postpairing phase (POST; 169 fecal samples) for an 8-week period following pairing. During both phases, marmosets were fed a consistent diet (commercial marmoset diet [Zupreem; Science Diet], *Tenebrio* larvae, scrambled eggs, fruits [red apple and cantaloupe], and gum arabic [Mazuri]). Fresh fecal samples were collected from marmosets in sterilized aluminum pans immediately after the light-on phase of the photoperiod. Samples were snap-frozen in liquid nitrogen and stored at −80°C. Most of these fecal samples were used in the previous gut microbiome study (1). This study was performed following the guidelines of the University of Nebraska Medical Center and the University of Nebraska at Omaha Institutional Animal Care and Use Committee. The protocol was approved by the University of Nebraska Medical Center/University of Nebraska at Omaha Institutional Animal Care and Use Committee (16-104).

### SCFA measurement.

Fecal samples were taken from the −80°C freezer and thawed at room temperature. Fecal samples (0.21 g) were homogenized with 1 ml sterile phosphate-buffered saline and then centrifuged (10,000 × *g*, 5 min). Next, 0.4 ml of supernatant from each sample was mixed with 0.1 ml of internal standard (7 mM 2-ethylbutyrate) followed by acidification, extraction into diethyl ether, and quantification by gas chromatography (Clarus 580; PerkinElmer, Waltham, MA, USA) based on the standard protocol (27). For each pair, we estimated two single average points, one from the PRE stage, the other from the POST stage (days after pairing: from 29 to 55). Wilcoxon paired tests were used to calculate the *P* value from these two groups (PRE versus POST) within each sex. The relationships between the abundance of the five gut microbiome genera (significantly increased in both females and males after pairing) and the concentration of PA were tested via linear and quadratic models. The better model was selected according to the lower value of Akaike’s information criterion (28). False discovery rates were addressed by the Benjamini-Hochberg procedure (B-H correction, α = 0.05) for multiple-comparison testing within each sex (29). The above statistical analysis and plotting were conducted in R software (30).
